# Modulation of Sirtuin 3 by N-Acetylcysteine Preserves Mitochondrial Oxidative Phosphorylation and Restores Bisphenol A-Induced Kidney Damage in High-Fat-Diet-Fed Rats

**DOI:** 10.3390/cimb46050296

**Published:** 2024-05-18

**Authors:** Anongporn Kobroob, Sirinart Kumfu, Nipon Chattipakorn, Orawan Wongmekiat

**Affiliations:** 1 Division of Physiology, School of Medical Sciences, University of Phayao, Phayao 56000, Thailand; anongporn.ko@up.ac.th; 2 Cardiac Electrophysiology Research and Training Center, Department of Physiology, Faculty of Medicine, Chiang Mai University, Chiang Mai 50200, Thailand; sirinart.kum@cmu.ac.th (S.K.); nipon.chat@cmu.ac.th (N.C.); 3 Integrative Renal Research Unit, Department of Physiology, Faculty of Medicine, Chiang Mai University, Chiang Mai 50200, Thailand

**Keywords:** bisphenol A, kidneys, mitochondria, high-fat diet, N-acetylcysteine, oxidative stress

## Abstract

Bisphenol A (BPA) and high-fat diets (HFD) are known to adversely affect the kidneys. However, the combined effects of both cases on kidney health and the potential benefits of N-acetylcysteine (NAC) in mitigating these effects have not been investigated. To explore these aspects, male Wistar rats were fed with HFD and allocated to receive a vehicle or BPA. At week twelve, the BPA-exposed rats were subdivided to receive a vehicle or NAC along with BPA until week sixteen. Rats fed HFD and exposed to BPA showed renal dysfunction and structural abnormalities, oxidative stress, inflammation, and mitochondrial dysfunction, with alterations in key proteins related to mitochondrial oxidative phosphorylation (OXPHOS), bioenergetics, oxidative balance, dynamics, apoptosis, and inflammation. Treatment with NAC for 4 weeks significantly improved these conditions. The findings suggest that NAC is beneficial in protecting renal deterioration brought on by prolonged exposure to BPA in combination with HFD, and modulation of sirtuin 3 (SIRT3) signaling by NAC appears to play a key role in the preservation of homeostasis and integrity within the mitochondria by enhancing OXPHOS activity, maintaining redox balance, and reducing inflammation. This study provides valuable insights into potential therapeutic strategies for preserving kidney health in the face of environmental and dietary challenges.

## 1. Introduction

Bisphenol A (BPA, 2,2-bis-(4-hydroxyphenyl)propane) is a key monomer in the production of polycarbonate plastics, epoxy resin, and other polymeric materials. BPA-based polycarbonate is widely used in the packaging of food and drinks, medical devices, thermal paper, and dental compounds [1]. BPA-containing epoxy resins are commonly used as coatings and linings for food and beverage cans to prevent food or drink from coming into direct contact with the container material [2]. Human exposure to BPA mostly derives from BPA released in foods and beverages when BPA-containing products are exposed to high temperatures, acidic or alkaline compounds, high sodium chloride, and a substantial amount of vegetable oil [3].

Several lines of evidence point to the harmful effects of BPA on various organ systems [3,4]. Oxidative stress is recognized as a key initiator of BPA toxicity [5]. Mitochondria are the major source of reactive oxygen species (ROS) production, and disturbance of homeostasis within the mitochondria causes excessive ROS generation and leads to adverse effects on several regulatory mechanisms within the body [6,7]. Regarding the kidney, evidence has demonstrated that BPA can act directly on the mitochondria, causing disturbance of mitochondrial homeostasis [5]. Besides, previous studies have linked ROS and mitochondrial dysfunction to renal injury in experimental models of both acute kidney injury and chronic kidney disease [6,7].

In today’s fiercely competitive world, the need for haste has become indispensable in daily life. This has resulted in a drastic change in human eating habits. Most people are turning to fast food and/or western diets, resulting in higher fat consumption. A high-fat diet (HFD) has been well acknowledged for its negative impacts on human health. Long-term HFD consumption has been shown to produce renal lipotoxicity and impair kidney function [8,9]. Studies have shown that oxidative stress and mitochondrial dysfunction underlie the adverse consequences of HFD consumption [8,9].

Because of its lipophilic nature, BPA can accumulate in different human and animal tissues, compromising their physiological functions and exerting deleterious effects on health [3]. There has been a report that HFD influences the activity of BPA in that it accelerates the development of adult disease after BPA exposure [10,11]. Considering the widespread and prolonged human exposure to BPA, along with the contemporary dietary shift towards high-fat foods, it is intriguing to explore the renal outcomes of concurrent long-term exposure to BPA and HFD. Besides, since BPA and HFD are closely related to oxidative imbalance and mitochondrial impairment, it is possible that interventions to reduce oxidative stress and/or maintain mitochondrial homeostasis would be beneficial in coping with this complex condition.

N-acetylcysteine (NAC) stands out as a promising therapeutic candidate due to its safety profile, affordability, and multifaceted pharmacological properties. Approved by the FDA for treating acetaminophen overdose and serving as a mucolytic agent, NAC has garnered attention for its pleiotropic effects, including antioxidant, anti-inflammatory, and anti-apoptotic activities [12]. Moreover, it has demonstrated protective benefits against disorders associated with oxidative stress and mitochondrial dysfunction across various organ systems, including the kidneys [4,12,13]. Of particular interest is the evidence proving that NAC can act directly within the mitochondria, underscoring its potential in mitigating cellular damage [14]. Importantly, our previous study demonstrated that NAC is effective in protecting against BPA-induced renal deterioration in normal diet-fed rats [15].

Building on these data, we conducted our study to explore the renal consequences of prolonged exposure to BPA in conjunction with HFD consumption and assess the therapeutic efficacy of NAC in this context. The mechanisms associated with renal alterations and the action of NAC were also investigated. This research not only sheds light on the interplay between environmental toxins, dietary habits, and renal function but also paves the way for the protective and therapeutic opportunity of NAC to counteract the negative impact of BPA exposure and unhealthy dietary practices on kidney function.

## 2. Materials and Methods

### 2.1. Chemicals, Reagents, and Antibodies

All chemicals and reagents (unless otherwise stated) were of analytical grade and were purchased from Sigma-Aldrich^®^ (Merck KGaA, Darmstadt, Germany). Antibodies against AMP-activated protein kinase (AMPK), sirtuin 3 (SIRT3), superoxide dismutase 2 (SOD2), B-cell lymphoma 2 (Bcl-2), pro-caspase3, cleaved-caspase3, phospho-dynamin-like protein 1 at Ser616 (p-Drp1^Ser616^), mitofusin 2 (Mfn2), phospho-IκBα (p-IκBα), phospho-NFκB p65 (p-NFκB p65), tumor necrosis factor-alpha (TNF-α), and β-actin were purchased from Cell Signaling Technology (Danvers, MA, USA). Peroxisome proliferator-activated receptor gamma coactivator 1 alpha (PGC-1α), phospho-AMPK at Thr172 (p-AMPK^Thr172^), and interleukin-1beta (IL-1β) were obtained from Merck (Merck KGaA, Damstadt, Germany). Total OXPHOS antibody cocktail, acetylated SOD2 (Ac-SOD2), and Bcl-2-associated X protein (Bax) were purchased from Abcam (Cambridge, MA, USA).

### 2.2. Animals

Eighteen male Wistar rats (Nomura Siam International, Bangkok, Thailand) weighing 130–150 g were housed on a 12 h light/dark cycle at 24 ± 1 °C with free access to water and standard rat chow. One week of acclimatization was allowed before starting the experiment. All the study protocols were approved by the Institutional Animal Care and Use Committee at the Faculty of Medicine, Chiang Mai University (protocol number 56/2563) and conformed to the Guide for the Care and Use of Laboratory Animals of the National Research Council of Thailand.

### 2.3. Diet and Experimental Design

After assessment of baseline renal function, all rats were switched to a high-fat diet (59.28% calories from fat, Table S1) [16] over the 16-week study period. Three experimental groups (*n* = 6 each) with no differences in basal renal function were assigned for the investigation. Group 1 (vehicle-treated group: HFV) and Group 2 (BPA-treated group: HFBPA) received vehicle and BPA (50 mg/kg dissolved in corn oil), respectively, by daily gavage through to the end of experiments. Group 3 (BPA plus NAC-treated group: HFBPA+NAC) was made up of BPA-exposed rats that concurrently received NAC treatment (100 mg/kg, orally) for 4 weeks (starting from week 12 to week 16 of the experiment). The selected dose of BPA as well as NAC was based on a recent study showing the adverse health effects of BPA on the kidney and its protection by NAC in rats fed a normal diet (Table S2) [15]. Food and water intake, including body weight, were recorded daily. After the last treatment, 24 h urine samples were collected using metabolic cages. Blood samples and kidney tissues were subsequently taken under thiopental (Ceva Animal Health Ltd., Bangkok, Thailand) anesthesia for further analyses.

### 2.4. Determinations of Triglycerides and Cholesterol

Serum triglycerides and total cholesterol levels were determined using an automatic analyzer (Beckman Coulter, Inc., Brea, CA, USA). Triglycerides and total cholesterol in the liver were measured using colorimetric kits obtained from Abcam, Cambridge, UK (No. ab65336 and ab65390, respectively) according to the manufacturer’s instructions.

### 2.5. Assessments of Renal Function

Blood urea nitrogen (BUN), serum creatinine, creatinine clearance, and urine protein-to-creatinine ratio were used as an index of renal function. All biochemical determinations were performed using an automatic analyzer (Beckman Coulter, Inc., Brea, CA, USA). Creatinine clearance was calculated using the standard clearance formula.

### 2.6. Light Microscopic Studies

The neutrally buffered formalin-fixed kidney tissues were routinely processed and embedded in paraffin. Tissue sections (4 μm thick) were stained with Hematoxylin and Eosin (H&E) using a standard histological staining procedure. Kidney slides were examined under a Leica DM750 photomicroscope (Leica Microsystems, Heerbrugg, Switzerland).

### 2.7. Transmission Electron Microscopic Studies

The electron microscopic studies were performed as previously reported [4]. Briefly, small fragments of renal cortical tissues were fixed in 2.5% glutaraldehyde, post-fixed in 2% phosphate-buffered osmium tetroxide, dehydrated through a graded ethanol series, and embedded using the EMbed-812 embedding kit (Electron Microscopic Sciences, PA, USA). Serial ultrathin sections (60–80 nm) were collected on copper grids, stained with uranyl acetate followed by lead citrate, and examined using a JEM-2200 FS transmission electron microscope (JEOL, Tokyo, Japan).

### 2.8. Assessments of Renal Oxidative Stress

Cayman’s TBARS Assay Kit (Cayman Chemical, Ann Arbor, MI, USA) and QuantiChrom™ Nitric Oxide and Glutathione Assay Kits (Bioassay Systems, Hayward, CA, USA) were used for assaying malondialdehyde (MDA), nitric oxide (NO), and glutathione (GSH), respectively, in kidney homogenates as per the manufacturer’s specifications.

### 2.9. Mitochondrial Studies

#### 2.9.1. Mitochondrial Isolation

The kidney cortex was homogenized in cold lysis buffer containing 230 mM mannitol, 70 mM sucrose, 1 mM EDTA, and 10 mM Tris-HCl, pH 7.4. Mitochondria were isolated by differential centrifugation as described previously [5] and resuspended in cold respiration buffer containing 250 mM sucrose, 5 mM KH_2_PO_4_, 10 mM Tris-HCl, 2 mg/mL BSA, and pH 7.2. Quantification of mitochondrial protein content was performed using a bicinchoninic acid (BCA) assay.

#### 2.9.2. Measurement of Mitochondrial Reactive Oxygen Species (ROS) Production

The cell permeant reagent 2′,7′-dichlorofluorescin diacetate (DCFDA) was used to quantify mitochondrial ROS production as previously described [5]. After staining mitochondria with DCFDA for 60 min, the emission of a highly fluorescent 2′,7′-dichlorofluorescein (DCF) was detected by fluorescence microplate reader (SynergyTM H4, BIOTEK^®^ Instruments, Inc., Winooski, VT, USA) with excitation/emission at 485 nm/535 nm. The extents of mitochondrial ROS production were expressed as arbitrary units of DCF fluorescent intensity.

#### 2.9.3. Measurement of Mitochondrial Membrane Potential

JC-1, a cationic carbocyanine dye that accumulates in energized mitochondria, was used to evaluate the change in mitochondrial membrane potential by the procedure described previously [5]. JC-1 shows potential-dependent accumulation in mitochondria, that is, green fluorescent monomers at low membrane potential and red fluorescent J aggregates at high membrane potential. After staining mitochondria with JC-1 for 30 min, the green monomer and the red J-aggregate form of JC-1 were detected by a fluorescence microplate reader using an excitation/emission of 485/530 nm and 485/590 nm, respectively. A decrease in the red/green fluorescence intensity ratio denotes mitochondrial depolarization.

#### 2.9.4. Measurement of Mitochondrial Swelling

The swelling of mitochondria was evaluated by the light-scattering technique as previously described [5]. The change in mitochondrial absorbance at 540 nm was monitored every 1 min for 15 min using a microplate reader (SynergyTM H4, BIOTEK^®^ Instruments, Inc., Winooski, VT, USA). Mitochondrial swelling was shown by a decrease in absorbance.

### 2.10. Western Blot Analysis

Western blotting was carried out to study the renal cortical expressions of total OXPHOS (complex I–V), AMPK, p-AMPK^Thr172^, PGC-1α, SIRT3, AC-SOD2, SOD2, pDrp1, Mfn2, Bax, Bcl-2, pro-caspase 3, cleaved-caspase 3, p-IκBα, pNF-κB p65, TNF-α, IL-1β, and β-actin (as loading control). Total protein was extracted from renal cortical tissues using lysis buffer (20 mM Tris-HCl, pH 6.8, 1 mM sodium orthovanadate, and 5 mM sodium fluoride) containing protease inhibitors, and protein concentration was determined by a Bradford protein assay kit (Bio-Rad Laboratories, Hercules, CA, USA). Total proteins were resolved on 10% SDS polyacrylamide gel electrophoresis (SDS-PAGE) and transferred onto nitrocellulose membranes (Thermo Fisher Scientific, Waltham, MA, USA). The membranes were blocked with 5% bovine serum albumin (BSA) or nonfat dried milk in Tris-buffered saline and Tween (TBST) and incubated overnight at 4 °C with the primary antibody specific to the protein of interest (Table S3), followed by the corresponding horseradish peroxidase (HRP)-conjugated second antibody at room temperature for 1 h. Clarity ECL Western blotting substrate kits (Bio-Rad Laboratories, Hercules, CA, USA) were used for detection of proteins bound to western blotting membranes, and the Image J program (version 1.54d) (National Institute of Health, Bethesda, MA, USA) was used for quantitative analysis of protein expression.

### 2.11. Statistical Analysis

The results are presented as means ± SEM. All data were analyzed for any differences between the groups using a one-way ANOVA followed by a Fisher post hoc test or a nonparametric Kruskal–Wallis test (as appropriate) with the use of SPSS software version 25 (IBM Corporation, Armonk, NY, USA). Statistical significance was set at *p* < 0.05.

## 3. Results

### 3.1. Effects of NAC Treatment on Metabolic Alterations after Long-Term BPA Exposure along with HFD Consumption

Table 1 shows metabolic alterations in all studied groups. Body weight, serum triglycerides, and total cholesterol levels at the start of the experiment were similar in all groups studied. At the end of the experiment, all HFBPA-treated groups, irrespective of NAC treatment, showed significantly lower body weights than those of the HFV-treated group. This occurred even though the total caloric intake was comparable between the groups. A similar trend was also observed in the kidney weights; however, there were no significant differences between groups in terms of the kidney weight to body weight ratio. Regarding triglycerides and total cholesterol levels, significant increases in serum triglycerides and total cholesterol were detected in all study groups after 16 weeks of HFD challenge. Surprisingly, these values were not significantly different between groups. We suspected that some triglycerides and cholesterol may accumulate in the liver, causing no difference in the serum levels between the studied groups. So, we measured liver triglyceride as well as cholesterol content, and, as expected, we found that triglyceride and cholesterol contents were dramatically increased in the liver of the HFBPA group compared to the HFV, and these changes were significantly attenuated by treatment with NAC.

### 3.2. Effects of NAC Treatment on Renal Function and Histopathology after Long-Term BPA Exposure Combined with HFD Consumption

**Figure 1:**
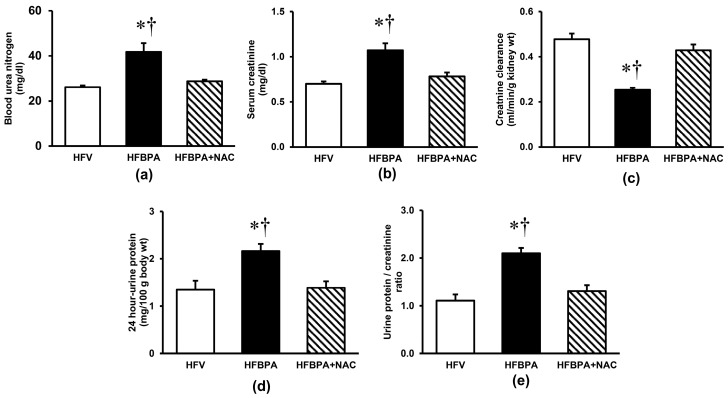
Effects of NAC treatment after long-term exposure to HFD and BPA on renal function. (**a**) Blood urea nitrogen; (**b**) Serum creatinine; (**c**) Creatinine clearance; (**d**) 24 h-urine protein; and (**e**) Urine protein/creatinine ratio. Values are means ± S.E.M (*n* = 6 each). HFV: HFD-fed rats treated with vehicle; HFBPA: HFD-fed rats treated with BPA; HFBPA+NAC: HFD-fed rats treated with BPA and NAC. * *p* < 0.05 vs. HFV, ^†^ *p* < 0.05 vs. HFBPA+NAC.

BPA exposure in combination with HFD consumption for 16 consecutive weeks resulted in significant increases in blood urea nitrogen and serum creatinine while decreasing in creatinine clearance (Figure 1a–c, respectively) compared with those of HFD feeding received only. A remarkable increase (*p* < 0.05) in 24 h-urine protein excretion was also evident in the HFBPA group (Figure 1d). The calculation of the urine protein-to-creatinine ratio (Figure 1e) exhibited a similar trend to the 24 h-urine protein. Interestingly, all renal functional alterations caused by the combination of long-term BPA exposure and HFD feeding were significantly restored when NAC was administered concomitantly.

**Figure 2:**
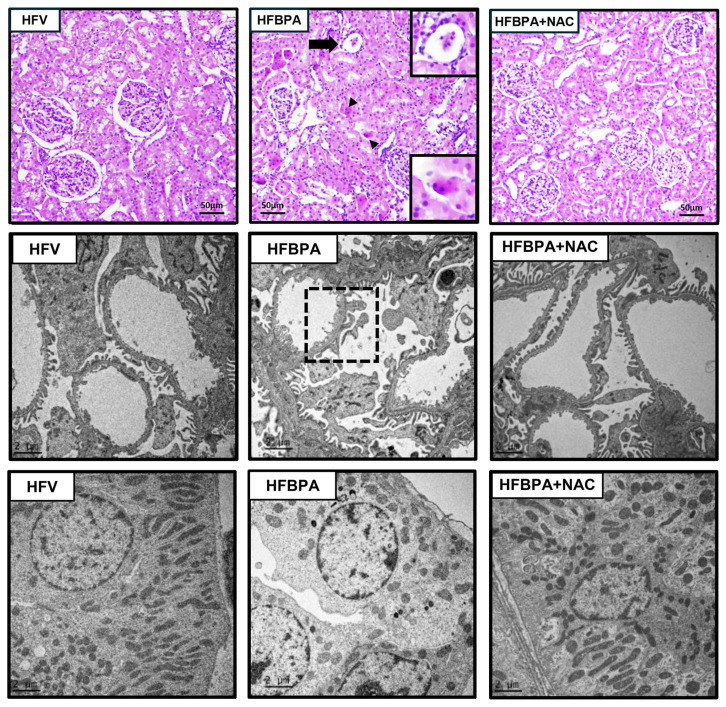
Effects of NAC treatment after long-term exposure to HFD and BPA on renal histopathology. The upper panel shows kidney sections stained with hematoxylin and eosin (H&E, 10×). The middle and lower panels show transmission electron micrographs of the glomerulus and proximal tubule, respectively (Original magnification: 3000×). HFV: HFD-fed rats treated with vehicle; HFBPA: HFD-fed rats treated with BPA; HFBPA+NAC: HFD-fed rats treated with BPA and NAC. Arrowhead shows apoptotic cells, arrow indicates glomerular atrophy, and dashed square demonstrates podocyte effacement.

Hematoxylin and Eosin staining of kidney sections (Figure 2, upper panel) in the HFBPA group showed a decrease in the number and size of glomerular tufts, glomerular atrophy, and diffuse apoptotic cells. Consistent with light microscopy, the electron microscopic images of the HFBPA-treated rats revealed thickening of the glomerular basement membrane and podocyte foot process effacement (Figure 2, middle panel). The proximal tubular cells of the HFBPA group also exhibited mitochondrial abnormal morphologies, including swelling, fragmentation, and a profound reduction in the number of mitochondria (Figure 2, lower panel). All these changes were more severe than those seen in the HFV group and were clearly alleviated with NAC treatment.

### 3.3. Effects of NAC Treatment on Renal Oxidative Stress and Mitochondrial Function after Long-Term BPA Exposure Combined with HFD Consumption

**Figure 3:**
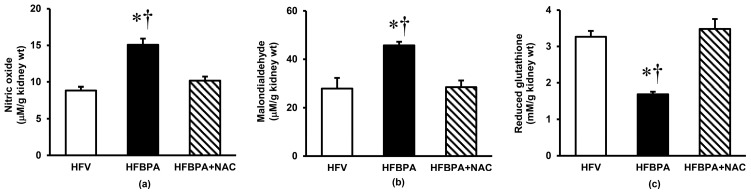
Effects of NAC treatment after long-term BPA exposure combined with HFD consumption on renal oxidative stress (**a**) Nitric oxide, (**b**) Malondialdehyde, and (**c**) Glutathione. Values are means ± SEM (*n* = 6 each). HFV: HFD-fed rats treated with vehicle; HFBPA: HFD-fed rats treated with BPA; HFBPA+NAC: HFD-fed rats treated with BPA and NAC. * *p* < 0.05 vs. HFV, ^†^ *p* < 0.05 vs. HFBPA+NAC.

The kidney tissue levels of nitric oxide (Figure 3a) and malondialdehyde (Figure 3b) after 16 weeks of HFD feeding along with repeated exposure to BPA were significantly higher than those received from HFD feeding alone. In contrast, the HFBPA-treated rats showed an almost two-fold decrease (*p* < 0.05) in the level of kidney glutathione (Figure 3c) compared to the HFV-treated rats. Treatment with NAC for 4 weeks was able to restore the changes in renal oxidative parameters induced by HFD and BPA.

**Figure 4:**
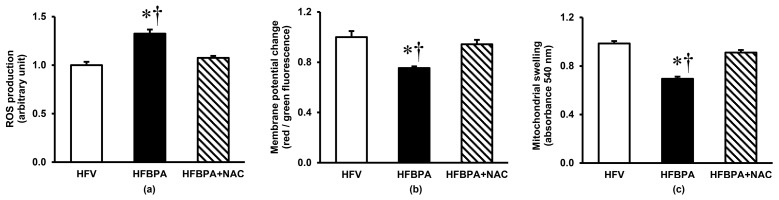
Effects of NAC treatment after long-term BPA exposure combined with HFD consumption on kidney mitochondrial function (**a**) ROS production, (**b**) Membrane potential change, and (**c**) Swelling. Values are means ± SEM (*n* = 6 each). HFV: HFD-fed rats treated with vehicle; HFBPA: HFD-fed rats treated with BPA; HFBPA+NAC: HFD-fed rats treated with BPA and NAC. * *p* < 0.05 vs. HFV, ^†^ *p* < 0.05 vs. HFBPA+NAC.

Regarding mitochondrial function, there was a considerable increase (*p* < 0.05) in mitochondrial ROS production by some 40% in the HFBPA-exposed group compared with the HFV group (Figure 4a). Disruption of mitochondrial membrane potential was apparent in mitochondria isolated from rats in the HFBPA group, being significantly lower than that observed in the HFV by some 30% (Figure 4b). Long-term exposure to HFD and BPA also induced mitochondrial swelling, as shown by a significant decrease in the absorbance of mitochondria at 540 nm (Figure 4c). Mitochondrial functional changes were prevented when NAC was administered simultaneously with HFD and BPA (*p* < 0.05).

### 3.4. Effects of NAC Treatment on Mitochondrial OXPHOS Protein Expression after Long-Term BPA Exposure Combined with HFD Consumption

**Figure 5:**
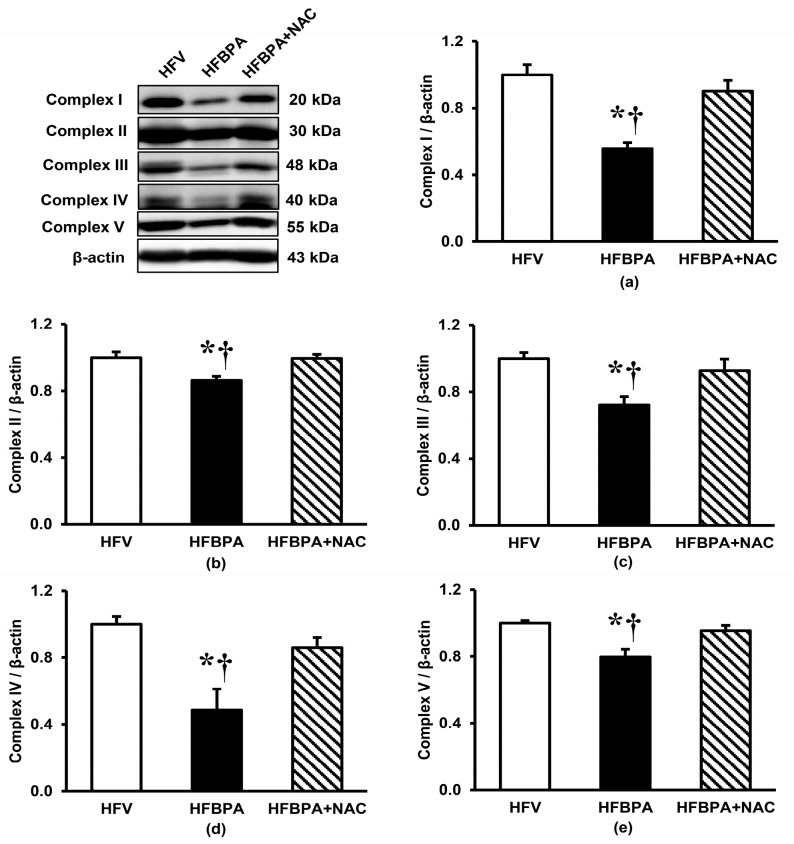
Effects of NAC treatment after long-term BPA exposure combined with HFD consumption on renal expression of mitochondrial oxidative phosphorylation (OXPHOS) proteins. (**a**) Complex I; (**b**) Complex II; (**c**) Complex III; (**d**) Complex IV; and (**e**) Complex V. β-actin was used as a loading control. Values are means ± SEM (*n* = 3 each). HFV: HFD-fed rats treated with vehicle; HFBPA: HFD-fed rats treated with BPA; HFBPA+NAC: HFD-fed rats treated with BPA and NAC. * *p* < 0.05 vs. HFV, ^†^ *p* < 0.05 vs. HFBPA+NAC.

The expressions of all the integral proteins for mitochondrial oxidative phosphorylation (i.e., complexes I–V) were markedly decreased (*p* < 0.05) in the HFBPA group compared to the HFV group (Figure 5). These changes were significantly improved in the group of HFBPA that was treated with NAC.

### 3.5. Effects of NAC Treatment on the Expressions of Signaling Proteins Involved in Mitochondrial Homeostasis after Long-Term BPA Exposure Combined with HFD Consumption

**Figure 6:**
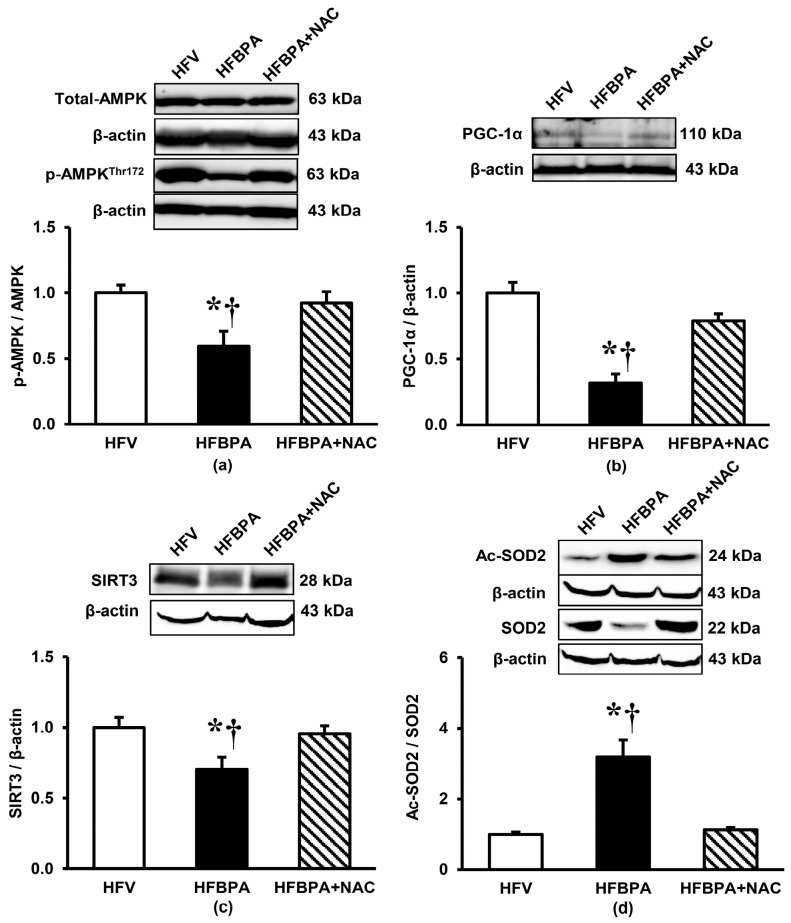
Effects of NAC treatment after long-term BPA exposure combined with HFD consumption on renal expression of signaling proteins involved in mitochondrial homeostasis (**a**) pAMPK/AMPK, (**b**) PGC-1α, (**c**) SIRT3, and (**d**) Ac-SOD2/SOD2. β-actin was used as a loading control. Values are means ± SEM (*n* = 3–4 each). HFV: HFD-fed rats treated with vehicle; HFBPA: HFD-fed rats treated with BPA; HFBPA+NAC: HFD-fed rats treated with BPA and NAC. * *p* < 0.05 vs. HFV; ^†^ *p* < 0.05 vs. HFBPA+NAC.

As shown in Figure 6, significant decreases in the protein levels of p-AMPK/AMPK ratio, PGC-1α, and SIRT3 were evident in the HFBPA group compared with those observed in the HFV group. In contrast, the HFBPA group showed a marked increase in the Ac-SOD2/SOD2 ratio compared to the HFV group (*p* < 0.05). Interestingly, all the changes seen in the HFBPA group were corrected when treated with NAC.

### 3.6. Effects of NAC Treatment on the Expression of Proteins Involved in Mitochondrial Dynamics and Apoptosis after Long-Term BPA Exposure Combined with HFD Consumption

**Figure 7:**
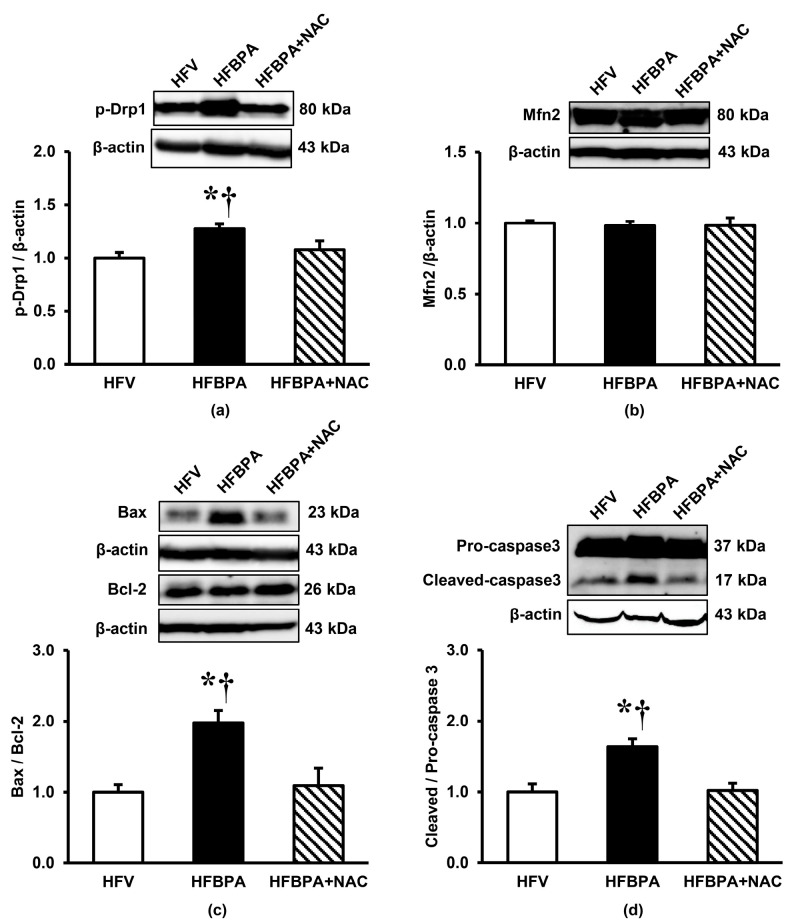
Effects of NAC treatment after long-term BPA exposure combined with HFD consumption on renal expression of proteins involved in mitochondrial dynamics and apoptosis. (**a**) p-Drp1, (**b**) Mfn2, (**c**) Bax/Bcl-2, and (**d**) Cleaved/Pro-caspase 3. β-actin was used as a loading control. Values are means ± SEM (*n* = 4 each). HFV: HFD-fed rats treated with vehicle; HFBPA: HFD-fed rats treated with BPA; HFBPA+NAC: HFD-fed rats treated with BPA and NAC. * *p* < 0.05 vs. HFV; ^†^ *p* < 0.05 vs. HFBPA+NAC.

The mitochondrial fission protein p-Drp1 was significantly increased in the renal cortex of the HFBPA group, while it was significantly reduced when NAC was administered concomitantly. However, there was no change in the expression of the mitochondrial fusion protein Mfn2 in both the HFBPA and HFBPA+NAC groups (Figure 7a).

Western blotting of the pro-apoptotic Bax was significantly increased in the HFBPA group, whereas the expression of the anti-apoptotic Bcl-2 remained unchanged. This led to a significantly higher Bax/Bcl-2 ratio in the HFBPA when compared with the HFV (Figure 7b). NAC treatment in the HFBPA rats significantly decreased Bax protein and had no effect on Bcl-2 protein, resulting in a decrease in the Bax/Bcl-2 ratio to a level close to that of the HFV rats. Similar trends were also found in the ratio of cleaved-caspase 3/pro-caspase 3, which was significantly increased after exposure to HFD along with BPA for 16 weeks and was restored by NAC (Figure 7c).

### 3.7. Effects of NAC Treatment on the Expression of Proteins Involved in Renal Inflammation after Long-Term BPA Exposure Combined with HFD Consumption

**Figure 8:**
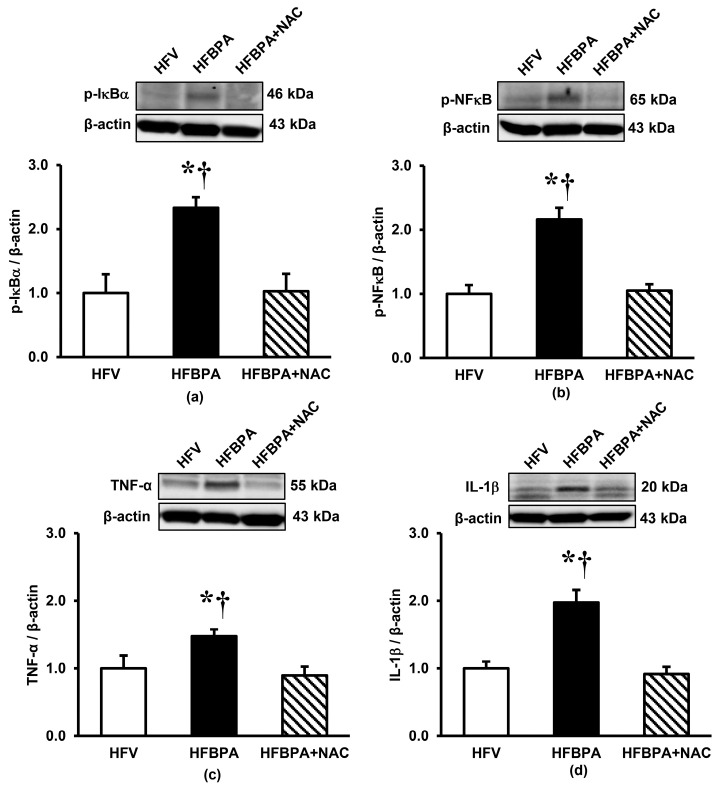
Effects of NAC treatment after long-term BPA exposure combined with HFD consumption on renal expression of proteins involved in inflammation (**a**) p-IκBα, (**b**) p-NFκB, (**c**) TNF-α, and (**d**) IL-1β. Values are means ± SEM (*n* = 4 each). HFV: HFD-fed rats treated with vehicle; HFBPA: HFD-fed rats treated with BPA; HFBPA+NAC: HFD-fed rats treated with BPA and NAC. * *p* < 0.05 vs. HFV; ^†^ *p* < 0.05 vs. HFBPA+NAC.

As compared to the HFV rats, the HFBPA rats showed significant increases in the protein expressions of p-IκBα and pNF-κB (Figure 8a,b). The protein levels of proinflammatory markers, TNF-α and IL-1β, were also significantly higher in the HFBPA group than the HFV group (Figure 8c,d). Treating the HFBPA rats with NAC was able to normalize these changes.

## 4. Discussion

This study focused on renal consequences after long-term BPA exposure combined with HFD consumption and, particularly, the therapeutic prospects of NAC. The study outcomes demonstrate that NAC effectively ameliorates renal dysfunction caused by long-term BPA exposure along with HFD consumption through its ability to modulate SIRT3 and thus maintain mitochondrial homeostasis and integrity.

BPA is a chemical that is produced worldwide in massive quantities each year for use in many common consumer products. Humans are regularly exposed to BPA from their daily activities and the adverse health effects of BPA are well accepted in various organ systems, including the kidney [4,15]. Long-term HFD consumption has also been well recognized as a risk factor for kidney disorders [8]. Given that BPA exposure is common and inevitable and that modern lives have changed to include more high-fat foods, it is very likely that both circumstances can coexist and become a serious problem that promotes kidney injury.

Here, we demonstrated that HFD feeding coupled with BPA exposure for 16 consecutive weeks leads to a deterioration of kidney function as signified by the retention of blood urea nitrogen and creatinine, the reduction in creatinine clearance, the presence of proteinuria, and the increase in urine protein-to-creatinine ratio. Both light and electron microscopies provided further support for the incidence of glomerular and proximal tubular damage. Our findings are in line with previous reports showing the unfavorable effects of HFD combined with chronic BPA exposure on the liver [17], reproductive system [18], cardiovascular, and metabolic systems [11,19]. Above all, the present investigation demonstrated that NAC treatment was effective in ameliorating renal dysfunction and minimizing the morphological changes brought on by HFD coupled with BPA.

Mitochondria are organelles that play a pivotal role in the control of homeostasis within the body. Mitochondria generate ATP to drive cellular functions through the oxidative phosphorylation (OXPHOS) process mediated by sequential electron transfer across the five membrane protein complexes (complexes I–V) of the electron transport chain (ETC). During the electron transfer, ROS are also generated, especially at complexes I and III. Thus, mitochondria are also the major source of ROS production, and mitochondrial dysfunction has been recognized as a hallmark of the initiation and progression of several diseases, including renal diseases [5,20,21].

In the present study, downregulation of the ETC complexes was noticed after prolonged BPA exposure combined with HFD consumption, which was accompanied by an increase in mitochondrial ROS production and oxidative stress in the kidney (evidenced by increased NO, MDA, and decreased glutathione). The findings suggested that simultaneous exposure to HFD and BPA impaired mitochondrial OXPHOS and induced ROS overproduction, which further increased the oxidative damage to various biomolecules not only in mitochondria but also in the whole kidney. Besides, increased mitochondrial oxidative stress could initiate a vicious cycle that further worsens mitochondrial function by advancing the damage to ETC components as well as other mitochondrial constituents, which led to fragmentation of the mitochondria, deterioration of OXPHOS, and exaggeration of ROS generation. This suggestion was substantiated by our results showing the loss of mitochondrial membrane potential after inhibition of electron transport due to a collapse of the proton gradient across the mitochondrial inner membrane. Moreover, our studies demonstrated that disruption of mitochondrial membrane potential induced the opening of mitochondrial permeability transition pore (mPTP, evidenced by mitochondrial swelling), mitochondrial fragmentation (demonstrated by increased p-Drp1/Mfn2 expression as well as electron microscopic images showing small size and reduced number of mitochondria), and cellular apoptosis (indicated by increased Bax/Bcl-2 and cleaved caspase3/pro-caspase3). Consistent with our findings, various experimental studies in several cell or tissue types have shown that BPA impaired mitochondrial bioenergetics and diminished ATP levels by uncoupling OXPHOS, inhibiting mitochondrial respiration, increasing oxidative stress, and thus altering mitochondrial morphology and function [1,21,22]. In view of HFD, there is also evidence that excessive consumption of nutrients affects the function of mitochondria. Studies have shown that long-term HFD intake leads to a high concentration of free fatty acids, which increases mitochondrial ROS production, impairs β-oxidation, and causes mitochondrial dysfunction [9,23]. The role of HFD-downregulated genes encoding proteins in complexes I, II, III, and IV of the electron transport chain has also been reported [24]. Collectively, it is indicated that mitochondrial oxidative stress and consequently mitochondrial dysfunction play a critical role in mediating renal injury following long-term HFD consumption coupled with BPA exposure in our study. Interestingly, all the changes caused by the effects of HFD and BPA were improved by NAC treatment. Our data pointed out that the recovery of kidney damages by NAC was related to a reduction in oxidative stress and the maintenance of mitochondrial homeostasis and integrity. At this point, we can only state that NAC may improve mitochondrial ETC function by attenuating the deleterious effects of oxidative stress in the kidney through its well-recognized free radical scavenging and antioxidant properties. This assumption is based on previous studies that demonstrate the beneficial effects of NAC supplementation in reducing HFD-induced metabolic disturbances [25], cardiovascular disorders [26], gut dysbiosis [27], hepatic steatosis [28], and neurodegeneration [29] by virtue of its antioxidant and anti-inflammatory properties. To explore other mechanisms related to the therapeutic efficacy of NAC, we further examined the expression of key proteins involved in the quality and quantity control of mitochondria.

SIRT3, a family of NAD^+^-dependent deacetylases, has been acknowledged to be involved in almost every aspect of mitochondrial metabolism and homeostasis, including protecting mitochondria from a variety of damage [30]. It localizes mainly to the mitochondrial matrix and is the major regulator of global protein acetylation in mitochondria [30]. SIRT3 plays a key role in the maintenance of mitochondrial redox homeostasis by regulating the functions of ETC complexes I, III, and V, thereby preventing excessive electron leakage and a consequent increase in ROS generation, hence powerfully boosting ATP levels [31,32,33,34]. SIRT3 also has a crucial role in the regulation of mitochondrial antioxidant systems and the detoxification of ROS through deacetylation and activation of SOD2 [33,34] in addition to maintaining reduced glutathione levels by directly activating isocitrate dehydrogenase 2 (IDH2) and increasing nicotinamide adenine dinucleotide phosphate (NADPH) levels [35].

Consistent with the above reports, we found that the expression of SIRT3 and SOD2 in the renal cortex of HFBPA-treated rats decreased, whereas the expression of Ac-SOD2 increased. These changes were related to an increased incidence of mitochondrial redox imbalance and were restored when treated with NAC. Further support for our findings is a previous study showing the correlation of SIRT3 activity with the maintenance of mitochondrial energy homeostasis and redox balance in the kidneys. It has been demonstrated that SIRT3 is highly expressed in the proximal tubule, where it regulates proximal tubular cell homeostasis by controlling microtubule network-dependent trafficking of functional mitochondria between renal tubular epithelial cells, a process that preserves the proper cellular bioenergetic profile and antioxidant defense [33]. Besides, there have been reports of a decrease in SIRT3 expression in the kidney following BPA exposure [4,15] as well as the worsening of kidney injury in SIRT3-deficient mice fed with HFD due to its high risk of oxidative stress and mitochondrial dysfunction [36]. Interestingly, a study using SIRT3 knockout mice has highlighted the role of SIRT3 in inhibiting the activity of cyclophilin D, the regulatory component of the mPTP complex, thus preventing the opening of mPTP and the loss of mitochondrial membrane potential, including mitochondrial fragmentation and death [34]. As loss of mitochondrial membrane potential, mitochondrial swelling, mitochondrial dynamics imbalance, and cellular apoptosis were also evident in our studies, all of which were ameliorated by NAC, it is most likely that activation of SIRT3 by NAC is a crucial mechanism behind the protection of HFBPA-induced mitochondrial dysfunction and renal damage in our study.

AMPK, a crucial cellular energy sensor responding to low ATP levels, plays a major role in the regulation of signaling pathways that replenish cellular ATP supplies. Upon changes in the ATP-to-AMP ratio, AMPK is activated and phosphorylates downstream targets to redirect metabolism towards increased catabolism and decreased anabolism. With respect to lipid metabolism, activation of AMPK reduces fatty acid synthesis and increases the uptake and oxidation of fatty acids by mitochondria [37,38]. In the present study, we also noticed that NAC significantly increased the expression of p-AMPK and restored a reduction in the ratio of p-AMPK/AMPK caused by HFD combined with BPA exposure. Based on these available data, it is likely that a further mechanism responsible for renal protection by NAC after HFD feeding combined with BPA exposure may lie in its ability to trigger AMPK phosphorylation and activation, which subsequently preserves mitochondrial homeostasis within the kidney. Corresponding to our findings, a decreased p-AMPK/AMPK ratio in the kidneys has been reported in long-term HFD-fed mice, which was linked to an increase in intracellular lipid content and mitochondrial dysfunction, and these adverse consequences were restored upon treatment with an AMPK activator [9,39]. A decreased p-AMPK expression and a decreased p-AMPK/AMPK ratio in the kidneys have also been observed after both short-term and long-term exposure to BPA [4,15].

In addition to its role in mitochondrial metabolic reprogramming, AMPK acts as a signal integration platform to maintain mitochondrial health by simultaneously regulating mitochondrial fission, mitophagy, and transcription control of mitochondrial biogenesis [40,41]. PGC-1α is a master regulator of mitochondrial biogenesis. AMPK signaling activates PGC-1α and, through a complex interplay between several key transcription factors, leads to the transcription of genes involved in the generation of new mitochondria [41]. In this study, we detected a decrease in PGC-1α expression as well as a reduced number of mitochondria in HFBPA-exposed rats (as seen by electron microscopy imaging), which were improved after treatment with NAC. The decreased PGC-1α in our study is presumably the result of a decrease in the ratio of p-AMPK/AMPK because PGC-1α is downstream of AMPK. Similarly, NAC boosted PGC-1α expression by raising p-AMPK/AMPK. Consistent with our results, downregulation of PGC-1α expression after long-term BPA exposure has been observed in the rat kidney [4,14] and heart tissues [42]. Additionally, suppression of PGC-1α expression following HFD feeding has previously been reported [43]. Interestingly, recent studies also suggest a role for PGC-1α in the regulation of OXPHOS, fatty acid/lipid metabolism, and the modulation of ROS [40]. All this information, together with our findings, supported that idea the restoration of PGC-1α derangement by NAC is involved in the alleviation of mitochondrial damage and renal injury following exposure to HFD combined with BPA.

In this study, we also detected increases in expressions of p-IκBα, p-NFκB, TNF-α, and IL-1β in the kidney after simultaneous exposure to HFD and BPA, all of which were decreased when treated with NAC. This corresponds to earlier studies showing an increase in ROS and proinflammatory cytokines following BPA administration as well as HFD consumption [1,44]. Although ROS is often considered the trigger of renal inflammation, the proinflammatory cytokines themselves can also induce oxidative stress. Our results suggested that renal inflammation also played a role in ROS generation and mitochondrial dysfunction under the conditions of HFD combined with BPA. The findings further indicated that NAC prevented upregulation of these proinflammatory cytokines by suppressing the activity of NF-κB. The anti-inflammatory effects of NAC via inhibition of NF-κB have been well established, in addition to its antioxidant activity [12]. Interestingly, SIRT3 has also been proven to inhibit inflammation by interfering with the NF-κB p65 subunit [45]. Moreover, there is increasing evidence that the PGC-1α-NF-κB/p65 interaction is an important hub in inflammatory pathways [40,41]. Evidence has shown that PGC-1α blocks NF-κB transcriptional activity toward its target genes, including those encoding pro-inflammatory cytokines [40]. In line with this information, it is suggested that activation of SIRT3, including increased expression of PGC-1α, by NAC may be another mechanism contributing to the anti-inflammatory actions of NAC in this study.

## 5. Conclusions

Emerging evidence from the current study reveals that NAC is helpful in protecting the kidney against the adverse consequences of long-term BPA exposure combined with HFD consumption. The mechanism underpinning the therapeutic potential of NAC in this complex situation is postulated to involve modifications of AMPK/PGC-1α/SIRT3 signaling, followed by maintenance of mitochondrial homeostasis and integrity. Given the broad impact of SIRT3 on mitochondrial metabolism and homeostasis, additional studies using knock-in and/or knock-out models of SIRT3 would be valuable for further exploration.

## Figures and Tables

**Table 1:** Metabolic alterations after long-term BPA exposure and NAC treatment in HFD-fed rats.

Parameters	Week 0			Week 16		
	HFV	HFBPA	HFBPANAC	HFV	HFBPA	HFBPANAC
BW (g)	134.17 ± 3.27	134.17 ± 2.39	135.00 ± 1.83	558.33 ± 13.76 ^‡^	427.50 ± 15.04 *^‡^	435.83 ± 9.87 *^‡^
Caloric intake (kcal/day)	-	-	-	92.85 ± 1.25	89.99 ± 1.45	90.86 ± 0.53
KW (g)	-	-	-	2.58 ± 0.06	2.00 ± 0.06 *	2.05 ± 0.07 *
KW/BW(×100)	-	-	-	0.47 ± 0.01	0.46 ± 0.01	0.47 ± 0.01
Serum TG (md/dL)	64.18 ± 6.43	65.21 ± 5.96	65.11 ± 5.69	78.82 ± 6.23	103.22 ± 9.43 ^‡^	86.69 ± 6.31 ^‡^
Serum TC (mg/dL)	100.16 ± 5.50	99.63 ± 5.77	99.73 ± 6.28	126.36 ± 9.68 ^‡^	141.68 ± 10.21 ^‡^	131.62 ± 6.17 ^‡^
Liver TG (mg/g LW)	-	-	-	1203.07 ± 69.42	1717.64 ± 50.11 *^†^	1253.24 ± 29.54
Liver TC (mg/g LW)	-	-	-	407.16 ± 31.94	591.72 ± 26.64 *^†^	463.09 ± 42.13

Values are means ± SEM (*n* = 6 each). HFV: HFD-fed rats treated with vehicle; HFBPA: HFD-fed rats treated with BPA; HFBPA+NAC: HFD-fed rats treated with BPA and NAC; BW: body weight; KW: kidney weight; LW: liver weight; TG: triglycerides; TC: total cholesterol. * *p* < 0.05 vs. HFV, ^†^ *p* < 0.05 vs. HFBPA+NAC within the same week. ^‡^ *p* < 0.05 vs. their respective values in week 0.

## Data Availability

The datasets generated and/or analyzed during the current study are available from the corresponding author on reasonable request.

## Supplementary Materials

The following supporting information can be downloaded at: https://www.mdpi.com/article/10.3390/cimb46050296/s1. Table S1: Composition and energy content of the high-fat diet; Table S2: Composition and energy content of the normal diet; Table S3: The details of antibodies used in this study.
