# Supplementary material for: Modulation of Sirtuin 3 by N-Acetylcysteine Preserves Mitochondrial Oxidative Phosphorylation and Restores Bisphenol A-Induced Kidney Damage in High-Fat-Diet-Fed Rats
Source: Curr Issues Mol Biol. 2024 May 18;46(5):4935–50. doi: 10.3390/cimb46050296 (PMC11119914; doi:10.3390/cimb46050296)
Supplement: Supplementary file 1 [file cimb-46-00296-s001.zip › cimb-2981268-supplementary.pdf]

Supplementary materials

Table S1. Composition and energy content of the high-fat diet (Pratchayasakul et al., 2011).

Composition	High-fat diet		
	**g**	**Kcal**	**% Energy**
Carbohydrate	190.76	763.04	14.27
Protein	353.60	1414.40	26.45
Fat (Lard)	342.24	3080.16	57.60
Cholesterol	10.00	90.00	1.68
Vitamin and mineral mixture	85.19		
DL-Methionine	3.00		
Fiber	13.21		
Yeast powder	1.00		
Sodium chloride	1.00		
**Total**	**1000**	**5347.60**	**100**
**Metabolizable energy**	**5.35 Kcal/g**		

Pratchayasakul, W.; Kerdphoo, S.; Petsophonsakul, P.; Pongchaidecha, A.; Chattipakorn, N.; Chattipakorn, S.C. Effects of high-fat diet on insulin receptor function in rat hippocampus and the level of neuronal corticosterone. *Life Sci* **2011**, 88, 619-627. doi:10.1016/j.lfs.2011.02.003

Table S2 Composition and energy content of the normal diet (CP082G, Perfect Companion Group Company Limited, Chiang Mai, Thailand).

Composition	Normal diet (CP082)		
	g	Kcal	% Energy
Carbohydrate	495.30	1981.20	51.99
Fat	83.70	753.30	19.77
Protein	269.00	1076.00	28.24
Vitamin and mineral mixture	65.40	-	-
Fiber	34.30	-	-
**Total**	**947.70**	**3810.50**	**100**
**Metabolizable energy**	**4.02 Kcal/g**		

Table S3. The details of antibodies used in the study.

Antibodies	Dilution	Catalog Number	Provider
Total OXPHOS antibody cocktail (Complex I-V)	1:1000	ab110413	Abcam
Total AMPK	1:1000	2532	Cell Signaling
p-AMPK^{Thr172}	1:1000	07-681	Merck
PGC-1α	1:1000	AB3242	Merck
SIRT3	1:1000	5490S	Cell Signaling
Ac-SOD2	1:1000	ab218529	Abcam
SOD2	1:1000	13194	Cell Signaling
p-DRP1^{Ser616}	1:1000	4494	Cell Signaling
Mfn2	1:1000	9482	Cell Signaling
Bax	1:1000	ab289364	Abcam
Bcl-2	1:1000	4223	Cell Signaling
Pro-caspase3<br>Cleave-caspase3	1:1000	14220	Cell Signaling
p-IKBα	1:1000	2859	Cell Signaling
p-NFκB p65	1:1000	3033	Cell Signaling
TNF-1α	1:1000	13377	Cell Signaling
IL-1β	1:1000	AB1832P	Merck
β-Actin	1:1000	8457	Cell Signaling
